# Human Induced Pluripotent Stem Cells as a Disease Model System for Heart Failure

**DOI:** 10.1007/s11897-020-00497-5

**Published:** 2020-11-19

**Authors:** Anton Deicher, Timon Seeger

**Affiliations:** 1 Department of Internal Medicine III, University Hospital Heidelberg, INF 410, 69126 Heidelberg, Germany; 2 German Centre for Cardiovascular Research (DZHK), Partner Site Heidelberg/Mannheim, Heidelberg, Germany

**Keywords:** Heart failure, Human induced pluripotent stem cells, Cardiomyocytes, Disease model

## Abstract

**Purpose of Review:**

Heart failure is among the most prevalent disease complexes overall and is associated with high morbidity and mortality. The underlying aetiology is manifold including coronary artery disease, genetic alterations and mutations, viral infections, adverse immune responses, and cardiac toxicity. To date, no specific therapies have been developed despite notable efforts. This can especially be attributed to hurdles in translational research, mainly due to the lack of proficient models of heart failure limited translation of therapeutic approaches from bench to bedside.

**Recent Findings:**

Human induced pluripotent stem cells (hiPSCs) are rising in popularity, granting the ability to divide infinitely, to hold human, patient-specific genome, and to differentiate into any human cell, including cardiomyocytes (hiPSC-CMs). This brings magnificent promise to cardiological research, providing the possibility to recapitulate cardiac diseases in a dish. Advances in yield, maturity, and in vivo resemblance due to straightforward, low-cost protocols, high-throughput approaches, and complex 3D cultures have made this tool widely applicable. In recent years, hiPSC-CMs have been used to model a wide variety of cardiac diseases, bringing along the possibility to not only elucidate molecular mechanisms but also to test novel therapeutic approaches in the dish.

**Summary:**

Within the last decade, hiPSC-CMs have been exponentially employed to model heart failure. Constant advancements are aiming at improvements of differentiation protocols, hiPSC-CM maturity, and assays to elucidate molecular mechanisms and cellular functions. However, hiPSC-CMs are remaining relatively immature, and in vitro models can only partially recapitulate the complex interactions in vivo. Nevertheless, hiPSC-CMs have evolved as an essential model system in cardiovascular research.

## Introduction

Cardiovascular diseases remain the number one cause of death worldwide with heart failure being among the leading factors for morbidity and mortality [1, 2]. However, despite numerous investments and enormous motivation, treatment advances and development of new drugs have been significantly low. Major reasons for the scarcity of novel and specific therapeutic approaches can be contributed to significant weaknesses in adequate model systems for mechanistical molecular analysis and drug development as well as drug testing.

Despite the opportunity to investigate pathophysiological molecular mechanisms of heart failure in vivo*,* approaches developed from widely used rodent models only very rarely lead to clinical therapies [3]. Furthermore, deciphering molecular mechanisms leading to and driving heart failure using human cardiomyocytes is significantly hampered by the lack of primary samples, sufficient controls, and the inability to sustainably maintain the cells in vitro*,* especially for drug discovery and testing.

**Fig. 1:**
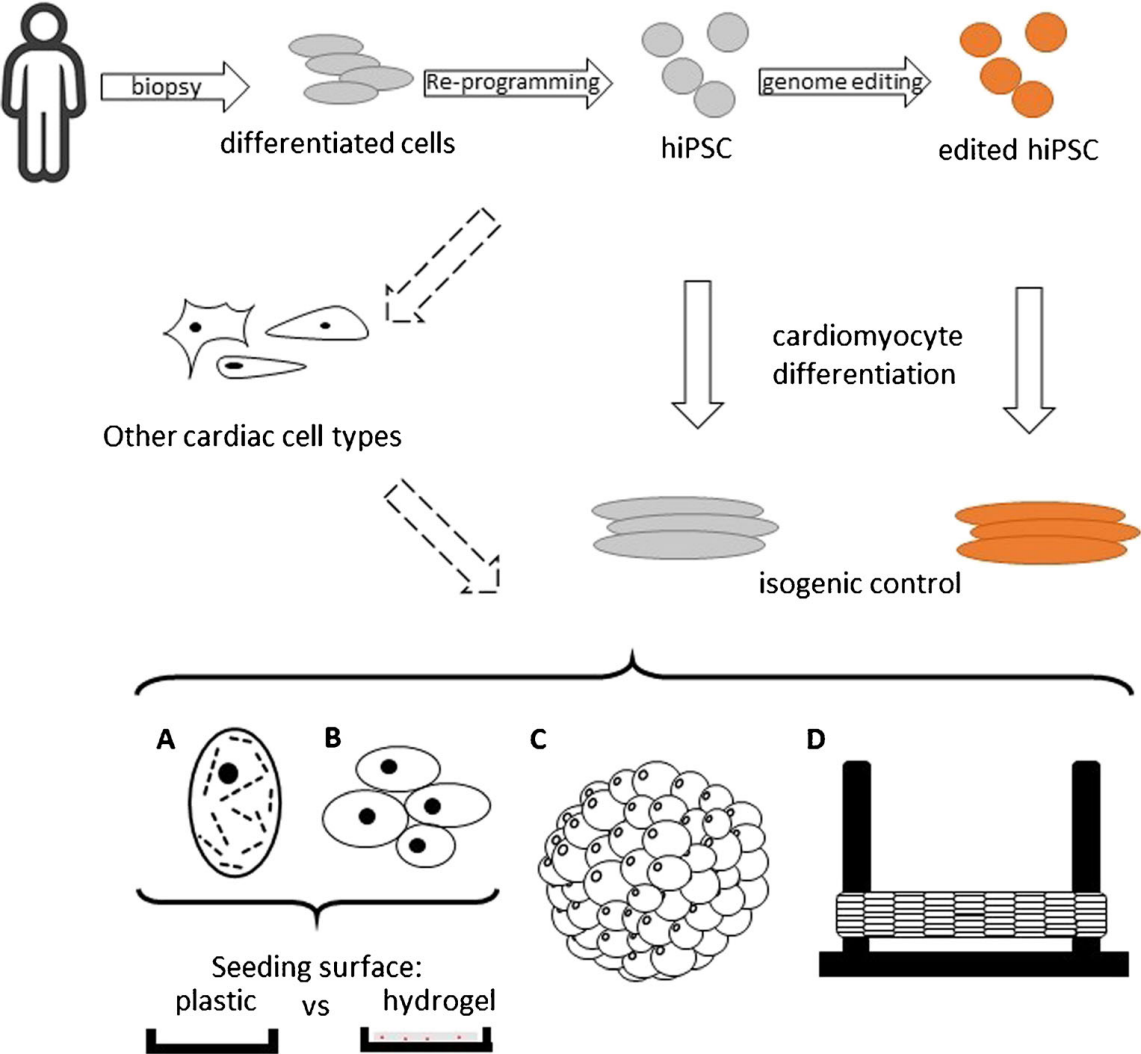
Approaches for the use of hiPSC-CMs as in vitro models. Primary samples (e.g. skin biopsies, peripheral blood cells, hair follicles) can be re-programmed to hiPSCs. Subsequently, genome-editing can be applied to generate isogenic hiPSC lines. Finally, hiPSCs can be differentiated into hiPSC-CMs using various protocols. The derived hiPSC-CMs can be further employed in assays for molecular, structural, and functional analysis. For this purpose, hiPSC-CMs can be seeded as (**a**) single cells or as (**b**) 2D clusters as well as monolayers, both on rigid plastic and more elastic surfaces (e.g. hydrogel-based preparations). 3D preparations include (**c**) unstructured 3D approaches, i.e. organoids, and (**d**) structured 3D culture, e.g. preparations between two rigid posts

In 2006, Takahashi et al. have presented a protocol to de-differentiate mature cells, generating human induced pluripotent stem cells (hiPSCs), thereby circumventing ethical concerns that come with using embryonic stem cells [4]. HiPSCs have the ability to differentiate into cells of all three germ layers, including cardiomyocytes, endothelial cells, smooth muscle cells, and cardiac fibroblasts. HiPSC-based models comprise several promises: Firstly, in contrast to primary cardiomyocytes gained by cardiac biopsy or post-mortem, cardiomyocytes from hiPSCs (hiPSC-CMs) can be generated unlimitedly. Secondly, hiPSC-derived cells are genetically identical to their donor, enabling disease-specific models in the scope of precision and personalized medicine. Thirdly, the combination of hiPSCs and genome-editing techniques comprises even more powerful applications for disease modelling by generating isogenic controls (see Fig. 1) [5, 6]. Lastly, models based on hiPSC-CMs can be used for drug discovery and cardiotoxicity screens.

During the recent years, hiPSC-based models have been implemented and further enhanced in order to understand developmental biology as well as pathophysiology and molecular mechanisms of various diseases in order to identify novel therapeutic strategies. However, insufficient maturation and prenatal physiology of hiPSC-derived cells as well as limitations in modelling the complex conditions in the beating human heart are still hampering broader applicability and translational relevance of hiPSCs-based models.

In this review, we outline the use of hiPSC-CMs as a disease model system for heart failure in cardiac research, describe methods for the analysis of hiPSC-CM phenotypes in different disease models for heart failure, and depict remaining challenges for this model.

## Generation of Cardiac Cell Types to Model Heart Failure

In the recent years, major improvements of differentiation protocols have led to the ability to reproducibly generate hiPSC-CMs either by monolayer or suspension culture-based approaches (as reviewed in Mummery et al. (2012) and Yoshida et al. (2017)) [7, 8]. The most widely used approaches for differentiation of cardiomyocytes from pluripotent stem cells have been derived from observations of cardiac development during embryogenesis targeting BMP4 and wingless-type mouse mammary tumour virus integration side (Wnt) pathways. Initially, differentiation of hiPSCs into hiPSC-CMs was dependent on embryoid body formation and has been subsequently simplified and standardized using monolayer-based protocols. Recently, major improvements have been achieved in upscaling the production of hiPSC-CMs, especially due to advancements in the development of suspension cultures in bioreactors [9].

A well-known limitation of hiPSC-CMs is their relative immaturity. To evaluate maturation, functional properties like a reduction of spontaneous beating activity, low resting membrane potential, intensified contractile force, positive force-to-frequency relationship, increase of contractile response to external calcium levels, and structural properties like the alignment of myofibres and mitochondria as well as the formation of t-tubules can be considered. On the molecular side, the ventricular isoform of myosin light chain (MLC2v) is highly expressed predominantly in adult ventricular cardiomyocytes and is therefore employed for the assessment of maturity [10•]. In addition, the expression of beta-adrenergic receptors (ADRB1/2), calveolin-3 (CAV3), ion channels (potassium: KCNJ2, calcium: CACNA1c), and troponin-T (TnnT2) is associated with mature cardiomyocytes in vivo [11].

Ongoing efforts are aiming at increasing maturity of hiPSC-CMs. A relative improvement in maturation by long-term culture of hiPSC-CMs was reported [12]; however, the functional phenotype is still more comparable to embryonic rather than postnatal cardiomyocytes. Further attempts to induce maturity in hiPSC-CMs include metabolic, electrical, and structural aspects. Metabolically, in vivo cardiomyocytes switch their diet from predominantly using glucose in embryonic stages to mainly utilizing fatty acids in matured stages. Cultivation of hiPSC-CMs in low glucose media in the presence of fatty acids has been shown to increase force generation, upstroke velocity, and expression of genes associated with mature cardiomyocytes [13]. Electrical stimulation has been shown to mature hiPSC-CMs, increasing polarity and connectivity and driving adult cardiomyocyte-specific gene expression especially in 3D preparations [10•]. Furthermore, hormones like triiodothyronine or dexamethasone were found to be able to improve calcium handling properties and lead to more structured organization of sarcomeres and a higher force generation in hiPSC-CMs [14, 15]. In terms of beneficial structural aspects, co-culture with fibroblasts [11], mesenchymal stem cells [16], or neural cells [17, 18] were revealed to improve hiPSC-CMs maturity. Finally, the 3D culture of cardiomyocytes can positively influence maturation in hiPSC-CMs (reviewed in Machiraju et al., 2019) [19].

Besides streamlining of differentiation procedures, multiple strategies have been developed to directionally differentiate hiPSCs into ventricular-like, atrial-like, and nodal-like subtypes of hiPSC-CMs [20, 21]. However, cardiac physiology and disease mechanisms are based on the interactions between cardiomyocytes and other resident cell types of the heart, like endothelial cells, fibroblasts, and smooth muscle cells. To date, several protocols have been published on the differentiation of these cell types from hiPSCs (reviewed in David et al., 2012 and Hausburg et al., 2017) [22, 23].

## Approaches to Model Heart Failure in a Dish Using hiPSC-CMs

In the recent years, a variety of technical concepts has been presented to utilize hiPSC-CMs as an in vitro model system for different aspects of cardiac diseases. This includes seeding hiPSC-CMs in various cell culture vessels and upon diverse substrates and surfaces as 2D monolayers, clusters, or single cells. Being able to generate large numbers of hiPSC-CMs, these cells can be used for higher-throughput approaches in multiwell plates containing up to 1536 wells. This allows for high-content screening, evaluating effects on functionality and viability using a variety of assays. However, compared to the beating human heart with its complex structural environment and constant mechanical and elastic changes, conceptually these techniques have their limitations. To recapitulate a more physiological environment, advances have been made to cultivate hiPSC-CMs on surfaces with reduced stiffness, for example, by using hydrogel formations [24]. This approach has also been shown to support long-term culture of hiPSC-CMs [25]. Furthermore, hiPSC-CMs can be cultured on elastic membranes, while cyclical stretching is applied, mimicking mechanical influences in the beating heart [26, 27].

Besides 2D monolayer approaches, hiPSC-CMs can also be cultured and analysed as single cells. Again, using multiwell approaches, the impact of large numbers of effectors on cellular characteristics, like cell size, intracellular structures, and intracellular trafficking among many others, can be evaluated in automated manners. Here, advancements in machine learning approaches will further broaden the abilities to establish specific readouts in cardiac research. Furthermore, single hiPSC-CMs can be micropatterned on substrates with defined stiffnesses in controlled sizes, e.g. representing the shape of cardiomyocytes in vivo with an estimation of a 7:1 length-to-width ratio [28]. This allows for detailed structural and functional analysis down to the level of the sarcomeric unit [29, 30].

Aiming at a more precise reflection of the situation in vivo, major achievements have been made in the generation of 3D constructs using hiPSC-CMs with and without other cardiac cell types (see Fig. 1). Initially, concepts were developed, based on synthetic filamentous matrices fixed with glass fibre [31]. Furthermore, re-population of extracellular matrix derived from decellularization of murine hearts has been shown to be effective in generating 3D structures (reviewed in Zia et al., 2016) [32]. To date, 3D cultures like engineered heart muscle (EHM) or engineered heart tissue (EHT) are mostly generated by solidification of defined cell/matrix solutions (e.g. on a fibrinogen or collagen basis) in moulds around posts of defined stiffnesses (Fig. 1), also holding the potential of force measurement deducted from post bending [11, 33]. Similarly, engineered cardiac tissue (ECT) was formed by using a net-like mould made of polydimethylsiloxane (PDMS) [34]. Also, scaffold-free methods such as cell sheet technologies produced by centrifugation of multiple cell layers or 3D printing were already applied to hiPSC-CMs [35]. In addition to hiPSC-CMs, defined proportions of other cardiac cell types can be added to further mimic intercellular interactions recapitulating an in vivo environment.

Besides engineered heart tissues, protocols have been presented to generate organoids including cardiomyocytes and other cardiac cell types based on various matrices (reviewed in Nugraha et al., 2019) [36]. Organoids are complex, unorganized, and self-assembled 3D structures mimicking organs in a dish, consisting of one or more cell types. These structures can recreate cell-cell as well as cell-matrix interactions and allow the cells to develop polarization [36]. Being less complex than engineered heart tissues, recent studies have used organoids as surrogates for cardiac 3D structures in high-throughput testing of cardiotoxicity [37, 38].

On physiological and also pathophysiological level, the crosstalk between cardiomyocytes and various other cell types, mainly endothelial cells (ECs), cardiac fibroblasts (CFs), and smooth muscle cells (SMCs), is crucial for maintaining homeostasis in the heart as well as for promotion of pathological processes [39, 40]. As described above, significant improvements of differentiation protocols are now enabling the generation of basically all cardiac cell types. Hence, it is possible to co-culture hiPSC-CMs with other cell types from the same hiPSC line and donor. Furthermore, ECs, CFs, and SMCs can be isolated and expanded from cardiac biopsies or unused donor hearts, and are commercially available, further allowing the employment of primary and mature human cardiac cells for co-culture with hiPSC-CMs. Methodologically, co-culture systems can be performed in 2D models directly or using permeable membrane systems for indirect communication without cell/cell contacts. Defined ratios of hiPSC-CMs and other cardiac cell types have also been presented for the generation of engineered heart tissues allowing for the evaluation of cell-cell communication in a more complex environment.

### Modelling Cardiac Diseases Leading to Heart Failure Using hiPSC-CMs.

Over the last few years, hiPSCs have been increasingly employed to elucidate pathophysiological mechanisms of various cardiovascular diseases (reviewed in Giacomelli et al., 2017) [41]. They have been shown to be effective for phenotype and drug response studies in diseases with genetic mutations such as long QT syndrome [42], short QT syndrome [43], catecholaminergic polymorphic ventricular tachycardia (CPVT) [44], atrial fibrillation [45], dilated cardiomyopathy (DCM) as well as hypertrophic cardiomyopathy (HCM) [5, 46], arrhythmogenic right ventricular dysplasia (ARVD) [47], Brugada syndrome [48], Timothy syndrome [49], and Duchenne muscular dystrophy [50]. Another area of application of hiPSC-based technologies has been the modelling of diseases with early-onset symptoms, in particular cardiometabolic diseases (Fabry’s disease [51], Pompe disease [52], Danon disease [53], Barth syndrome [54]). Beyond these, hiPSC-CMs are also analysed in order to identify disease-driving mechanisms, e.g. in hypoplastic left heart syndrome [55] and other congenital heart diseases.

## Analysis of hiPSC-CM Models

To model heart failure in hiPSC-CMs, structural and functional analysis can be performed to identify in vitro phenotypes. Constant advances in bioengineering and assay development are enhancing the utilization of heart failure models based on hiPSCs. Generally, establishing robust functional read-outs is essential to determine disease-relevant molecular mechanisms in high-content analysis approaches and ultimately can lead to identify novel therapeutic options.

### Structural Analysis of hiPSC-CMs.

Despite their relative immaturity, hiPSC-CMs inherit sarcomeric and subcellular organization and can be used for structural analysis. In recent studies, cardiac disease phenotypes reflected in hiPSC-CMs have been expressed in terms of sarcomeric disarray [56], multinucleation [57], and hypertrophy [58]. Generally, approaches include immunostaining, commonly targeting sarcomeric proteins like sarcomeric actinin, myosin light chain, or cardiac troponin T. However, also live-staining approaches using small molecules, viral transduction, or reporter hiPSC lines have already been used to evaluate structural changes over time [59].

With the advent of machine learning approaches and automated analysis tools, in-depth structural analysis can be performed way beyond simple estimation of 2D cell area. These high-content assays can help to identify structural disease-specific phenotypes in vitro, comparing responses of healthy versus diseased hiPSC-CMs to a variety of stressors (like catecholamines, hypoxia, or nutrient deprivation) or treatments (e.g. small molecule screens, gene silencing libraries, kinase inhibitor libraries). Vice versa, healthy hiPSC-CMs can be used in biomarker assays, e.g. to correlate effects of serum from cohorts of heart failure patients with the individual clinical courses. This approach can be applied to various other clinical conditions, therefore serving as a tool to identify patients at risk further promoting personalized medicine. In addition, novel drivers of disease can be discovered by analysis of patient serum components in comparison to cellular phenotypes of treated hiPSC-CMs.

### Contractility Analysis of hiPSC-CMs.

A major characteristic of hiPSC-CMs is their ability to electromechanically couple and contract. Contractility is of critical importance for heart function and a hallmark of heart failure when impaired. Recent studies employing hiPSC-CMs from patients with DCM have shown reduced contractile functions and limited force generation in these cells compared to healthy hiPSC-CMs [60]. Up to date, several concepts for contractility analysis exist depending on low- or high-throughput strategies as well as the capacity of bioengineered models of 2D and 3D systems to more closely reflect in vivo situations. In addition, contractility can be assessed comparing diseased and healthy hiPSC-CMs under various stress conditions as well as in response to clinical samples.

On single-cell level, contractile properties like sarcomeric shortening, cellular shortening, and single-cell force generation can be evaluated using high-resolution video microscopy [29, 30, 61]. Additionally, force generation can be measured by tracking the displacement of fluorescent particles in the substrate that the cells are plated upon (e.g. hydrogel formulations). Traction force microscopy is based on recording the distances these particles are moved within the substrate upon contraction and relaxation [62]. Besides the assessment of contractile function based on video microscopy, force of single cells can also be measured physically, for example, using atomic force microscopy [60].

In order to increase the analysis throughput, contractility can be assessed in 2D monolayers, either on regular plastic as well as glass bottom multiwell plates, or on surfaces with defined stiffness (with and without fluorescent microbeads). Two main strategies have been established: high-speed video-based microscopy and evaluation of contractility by tracking changes in electrical impedance.

**Fig. 2:**
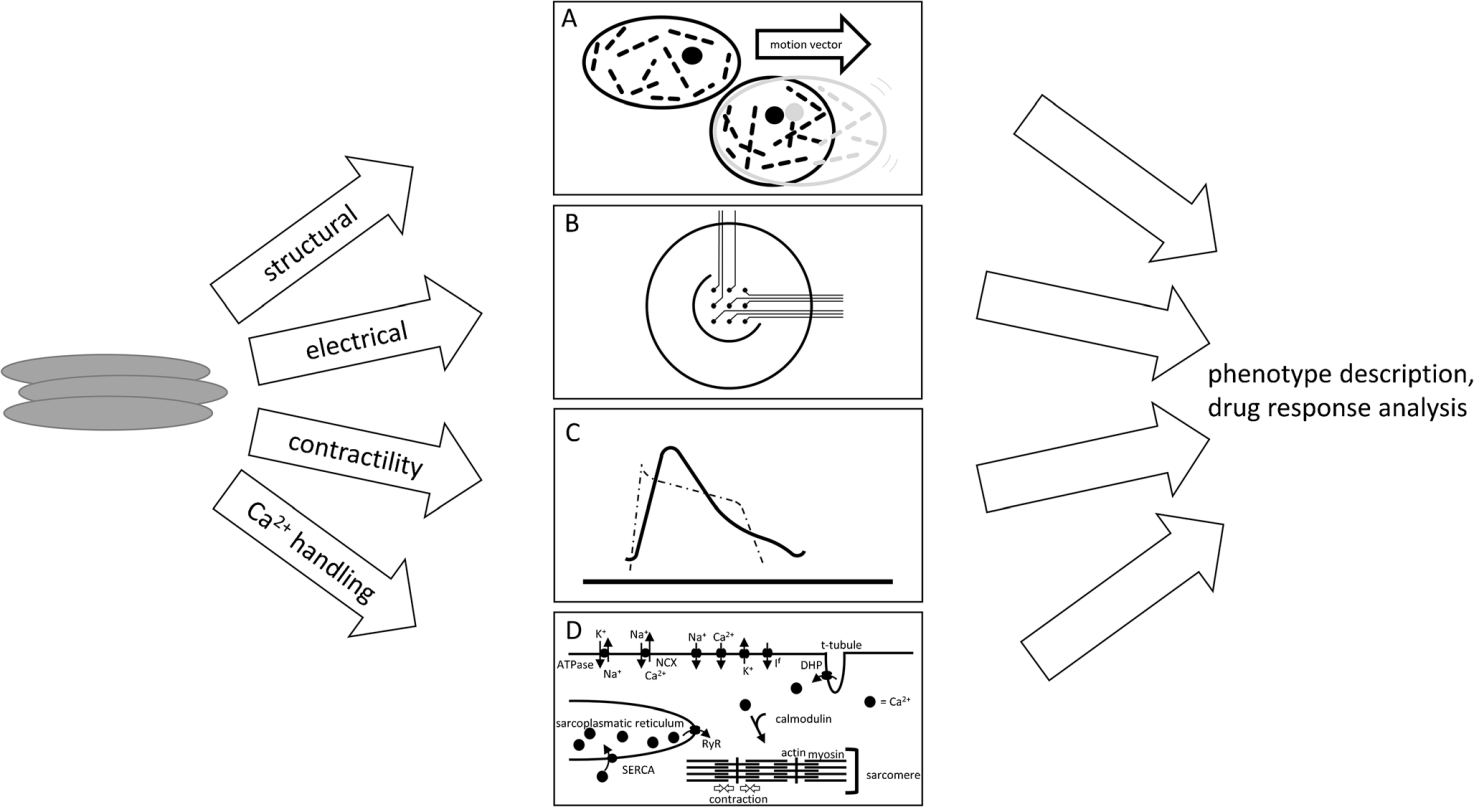
Modelling heart failure with hiPSC-CMs. Depiction of methods generating read-outs for analysis of disease models with hiPSC-CMs. **a** Singularized hiPSC-CMs can be used for structural analysis. Furthermore, contraction analysis of single hiPSC-CMs can be performed with video microscopy. **b** HiPSC-CMs can be seeded on plates with electrodes for multielectrode assay (MEA) analysis. **c** Graphic of contraction and action potential (dashed). Contractility assays of 2D monolayer approaches can be based on video microscopy or impedance measurement. Direct force generation can be measured in 3D culture approaches. **d** Illustration of proteins associated with calcium handling. Calcium handling properties can be measured using calcium-sensitive dyes

Using video recordings with high spatial and temporal resolutions, contractility is estimated based on motion vectors generated by tracing pixel movement (see Fig. 2) [63, 64]. Thereby, contraction and relaxation velocity as well as deformation distance can be determined, serving as surrogate parameters for force generation [62, 63, 65]. Due to its accessibility and unpretentious technique, this approach is widely used and scalable for approaches in multiwell plate formats in order to perform high-content screening assays using contractility as a readout. Additionally, traction force microscopy can be used by utilizing fluorescent microbeads incorporated in hydrogel in 2D monolayer approaches, as outlined above for single-cell analysis.

Besides video-based analysis, contractile function can be evaluated by recording changes in impedance during contraction and relaxation of hiPSC-CMs. For this purpose, hiPSC-CMs are seeded on plates with several electrodes embedded in the bottom of each single well, allowing for continuous and simultaneous recording of all wells, currently up to a format of 96-well plates (multielectrode analysis (MEA); see Fig. 2) [66]. The changes in impedance during contraction and relaxation correlate directly with contractile force generation in hiPSC-CMs [67]. However, while this approach enables simultaneous recording of all wells over long periods of time, the setup requires specific machines, and MEA plates are quite expensive due to the technical efforts in the production process.

Direct analysis of absolute force generation can be robustly performed using 3D structures like EHTs and EHMs, either from hiPSC-CMs alone or as a co-culture with other cardiac cell types, as outlined above. Using video microscopy, the moving distance of the posts carrying the EHTs/EHMs throughout contraction and relaxation can be recorded, and absolute force can be deducted. Furthermore, the passive stiffness can be calculated allowing for modelling of cardiac fibrosis and, to a certain extent, diastolic impairment [68, 69]. Recently, the implementation of magnetic particles in the tips of the posts has been reported, enabling simultaneous recording using a specially designed plate [70]. Furthermore, in a recent study modelling Barth syndrome, 3D analysis of contractility has been performed by generating sheets of hiPSC-CMs that are fixed on one end while bending the free end upon contraction. Video-based recording of the bending distance has been used in this study as a surrogate marker for contractility, revealing a contractile impairment in diseased hiPSC-CMs [71].

Generally, the evaluation of contractility is prone to variability due to various reasons. An accurate heating control for uniform temperature distributions throughout the plate and between measurements is crucial since already slight changes in temperature affect force generation [72]. When preparing hiPSC-CMs for seeding either in 2D or 3D applications, variability of differentiation efficiency, precise counting of the cells, differing viability after seeding of cells, and, especially in 3D applications, altering effective formation of a syncytium of simultaneously contracting cells can lead to significant variations in beat rate and contractility.

To reduce variability, beating of hiPSC-CMs in various preparations can be synchronized by electrical pacing in order to control beat rate. Moreover, continuous pacing has been shown to be beneficial regarding maturation in hiPSC-CMs [10•]. One major advantage of MEA plates is the inherent possibility to additionally utilize the electrodes for electrical pacing. However, long-term monophasic electrical pulses may cause cellular damage due to redox reactions at the site of the electrodes, which might be ameliorated using biphasic stimuli [73]. Over the past few years, optogenetics has been increasingly employed for pacing of cardiomyocytes [74]. Here, light-sensitive ion channels (e.g. channelrhodopsin-2) are introduced into hiPSC-CMs either by genetic insertion into the parental hiPSCs using genome editing or by viral transduction (e.g. adeno-associated virus-mediated transfer) [75]. Subsequently, stimulation can be achieved by periodically applying light in the specific wavelength that leads to activation of the ion channel employed. This method inherits significant potential due to the precision of the excitation at single-cell level both in high spatial and temporal resolution, as well as the possibility of coupling the expression of optogenetic channels with cell line-specific promoters. Furthermore, physical interaction with the cells is minimized, reducing in turn the risk of contamination.

Taken together various approaches exist to employ hiPSC-CMs for different aspects of heart failure research. So far, 2D monolayer approaches are most widely used because of their scalability as well as relatively low technical efforts. However, the use of 3D models is significantly increasing driven by advancements in preparation procedures. These approaches allow for more complex structures that more closely resemble in vivo conditions.

### Electrical Analysis of hiPSC-CMs.

Besides low cardiac output due to reduced contractility, patients with heart failure are threatened by malignant arrhythmias like ventricular tachycardia or fibrillation. As mentioned above, several studies have been reported using hiPSC-CMs to model channelopathies, namely long QT [42], short QT [43], and Brugada syndrome [48], but also arrhythmic diseases like CPVT [44]. Furthermore, several other genetically caused cardiomyopathies are associated with life-threatening arrhythmias like HCM [5], DCM [46], and ARVD [47]. Finally, beyond disease-inherent risks for arrhythmias, a majority of new therapeutic approaches, not only in cardiology, fail in clinical trials because of side effects resulting in malignant arrhythmias, mainly due to QT prolongation. Several approaches have been established to evaluate the electrical properties of hiPSC-CMs. Traditionally, cardiomyocytes can be analysed by patch-clamp methods, which are also broadly applied to hiPSC-CMs [76]. Here, single-channel analysis or general properties of the membrane potential can be recorded by targeting single cells or cells in small clusters. While patch-clamp methods are indispensable for the evaluation of cardiomyocytes when looking into channel properties and specific drug effects, this method is laborious and technically challenging, resulting in high-quality data but relatively low throughput. As a consequence, automated setups for patch-clamping have been commercially developed; however, they are not yet broadly used [77].

Aiming at higher throughput, hiPSC-CMs can also be seeded on MEA plates allowing for the recording of field and action potentials as well as propagation properties in 2D monolayer preparations besides the aforementioned analysis of impedance. As for impedance analysis, recordings can be performed in parallel and over time allowing for precise determination of disease and drug effects on the cells [78, 79].

Finally, using voltage-sensitive dyes, analysis of field and action potential can be performed in 2D monolayers of hiPSC-CMs using video microscopy [74, 80]. In principle, this approach can also be applied to 3D constructs.

However, while scalable, MEA and voltage-sensitive dye-based approaches can only record electrical activity as a summation of all cells in the syncytium either above one electrode or within the field of view in contrast to patch-clamping analysis in single hiPSC-CMs.

### Analysis of Calcium Handling Properties in hiPSC-CMs.

Electromechanical coupling in cardiomyocytes is significantly mediated by calcium. Calcium is released from the sarcoplasmic reticulum upon depolarisation binding to sarcomeric proteins, ultimately resulting in contraction. Finally, calcium is pumped back into the sarcoplasmic reticulum at the end of the contraction cycle resulting in relaxation of the myofilaments. Pathophysiological changes in intracellular calcium levels and altered sensitivity of the sarcomeres to calcium are directly related to cardiac disease phenotypes [81, 82]. Several drugs have been developed aiming at increasing calcium sensitivity or cellular calcium levels in the setting of heart failure [83]. However, while conceptually intriguing, none of these approaches has resulted in broad clinical applicability due to increased cellular energy expenditure levels and higher risk of ischemia with increased incidents of arrhythmia, leading to increased mortality [83].

HiPSC-CMs can also be utilized to assess disease-relevant changes in calcium handling properties in vitro or to evaluate the efficiency of new therapeutic approaches. For calcium analysis, hiPSC-CMs can be stained with non-toxic calcium binding fluorescent dyes. Generally, analysis can be performed using hiPSC-CMs in single cells, 2D cell clusters, or monolayers but also in 3D constructs. Upon intracellular release of calcium, these dyes, when excited by light in specific wavelengths, emit light that can be recorded by video microscopy. Many dyes like fluo-4 need only one wavelength, enabling the analysis of multiwell plates using high-content screening machines similar to 2D monolayer approaches analysing contractility [74, 84]. However, the obtained output only displays relative changes of intracellular calcium levels, not allowing for the assessment of diastolic levels. This can be achieved by using ratiometric approaches with dyes like fura-2 [74]. Furthermore, calcium signalling has been used to assess the risk of arrhythmogenic potential based on the analysis of calcium sparks that can be detected as spontaneous intracellular calcium release [84].

### Further Evaluation of Cellular Functions of hiPSC-CMs.

Beyond the analysis of functional properties of hiPSC-CMs like contractility, electrical activity, or calcium handling, general cellular functions like apoptosis, metabolism, and mitochondrial capacity can be assessed. Drug-related cardiotoxicity can regularly result in heart failure due to direct cytotoxic effects [85]. In 2D monolayer settings, assays depicting apoptosis can be used in multiwell approaches to further quantify cardiotoxicity. This includes evaluation of histone-associated DNA [86] or labelling of DNA strand breaks (TUNEL staining) [87], and evaluation of signalling pathways, like Caspase assays [88], or assessing apoptosis with the usage of flow cytometry (e.g. based on Annexin A5 labelling [89]). Furthermore, plate reader assays evaluating, for example, cellular adenosintrisphosphate (ATP) levels to analyse energy metabolism as an indicator for cell viability or assessment of reactive oxygen species (ROS) to evaluate cellular stress, can be performed using 2D monolayer preparations of hiPSC-CMs [16]. These approaches are scalable in multiwell assays up to 384- or even 1536 well plates for high-content screening, e.g. in cardiotoxicity screens [88]. For specific bioenergetical insight into hiPSC-CMs, complex assays for real-time intracellular oxygen and proton levels can be performed to evaluated potential disease-related phenotypes as well as responses to drugs or various stress conditions [90].

## Challenges and Conclusion

The advent of hiPSC-based technology comprises significant potential to cardiovascular research, especially in regard to elucidating mechanisms in heart failure and developing new and specific therapeutic approaches. To date, hiPSCs have been used to model a wide variety of cardiac diseases, including channelopathies, sarcomeric aberrations, and metabolic diseases. To establish in vitro disease phenotype, assays for contractile, electrical, structural, and general cellular functional properties are available. However, there are still several complexities to encounter. Similar to the variability in comparing human individuals, hiPSC lines vary greatly due to their genetic background. Furthermore, aspects like epigenetic memory from parental cells contribute to various extends to the variability intrinsic to pluripotent stem cells, leading to complex reproducibility [91]. In addition, variability not only between different hiPSC lines but also between different batches of differentiation and among laboratories has not yet been fully resolved. Reproducible results are also impacted by highly sensitive responses of hiPSC-CMs to changes in temperature, media, or gas levels and their reliance on time accuracy in the execution of most differentiation protocols. Ultimately, although hiPSC-CMs recapitulate various structural and functional aspects of adult cardiomyocytes in vivo, their state of maturity is significantly limited despite ongoing efforts.

The current standard of hiPSC-CMs culture is complex; however, with constant improvements in differentiation protocols, cell culture conditions, and assay development, this technology holds great promises for deciphering molecular mechanisms of heart failure as well as drug discovery. Especially advancements in 3D applications and in the context of organ-on-a-chip and body-on-a-chip concepts, combining various tissues in a shared microenviroment will significantly promote the use of hiPSC-technology in cardiovascular and specifically heart failure research.
